# Revisiting the Prominent Anti-Tumoral Potential of Pre-mNK Cells

**DOI:** 10.3389/fimmu.2013.00446

**Published:** 2013-12-11

**Authors:** Fanny Guimont-Desrochers, Sylvie Lesage

**Affiliations:** ^1^Immunology-Oncology Section, Maisonneuve-Rosemont Hospital, Montreal, QC, Canada; ^2^Département de Microbiologie, Infectiologie et Immunologie, Université de Montréal, Montreal, QC, Canada

**Keywords:** natural killer cells, cellular differentiation, pre-mNK cells, anti-tumoral activity, human, mouse

## Abstract

Interferon-producing killer dendritic cells (IKDC) were first described for their outstanding anti-tumoral properties. The “*IKDC*” terminology implied the description of a novel DC subset and initiated a debate on their cellular lineage origin. This debate shifted the focus away from their notable anti-tumoral potential. IKDC were recently redefined as precursors to mature NK (mNK) cells and consequently renamed pre-mNK cells. Importantly, a putative human equivalent of pre-mNK cells was recently associated with improved disease outcome in cancer patients. It is thus timely to revisit the functional attributes as well as the therapeutic potential of pre-mNK cells in line with their newly defined NK-cell precursor function.

## Introduction

The immune system forms an elaborate network of multiple cell types which together collaborate to eliminate unwanted pathogens and tumor cells. Among immune cells, natural killer (NK) cells demonstrate rapid cytotoxic activity upon the first signs of infections or cellular transformation (1, 2). The cellular particles and debris resulting from NK cell-mediated lysis are rapidly engulfed by the neighboring dendritic cells (DC) (3). DC are specialized antigen-presenting cells, which process and present antigens to T cells, thereby initiating the adaptive arm of the immune response (4). DC and NK cells are thus essential components of the innate immune system.

Interestingly, unique cell types conjugating properties of both NK cells and DC have previously been described (5, 6). In humans, activated NK cells have been reported to express MHC class II and present antigens to T cells (7–10). Conversely, in humans and in rodents, some DC subsets exhibit cytolytic activity (11–20). More recently, a murine cell type exhibiting properties of both NK and DC was identified and named interferon-producing killer dendritic cells (IKDC) (21, 22). As for NK cells, IKDC exhibit the capacity to produce IFN-γ and to mediate cytotoxic activity and, as for DC, they efficiently present antigens and stimulate T cell responses (21, 22). The combination of these unique properties endows IKDC with a unique immune potential allowing to specifically bridge functions of the innate and adaptive immune system. The outstanding biological potency of IKDC was rapidly noted in various *in vivo* tumoral settings (22–24). However, the study of IKDC for their anti-tumoral properties was rapidly halted as a debate emerged regarding both the cellular lineage origin of IKDC and their hybrid cellular properties (5, 25–29). IKDC have now been recognized as part of the NK-cell lineage (26–28, 30) and have since been renamed pre-mature NK (mNK) cells (30, 31). As the debate on the lineage origin comes to a close, the means by which pre-mNK cells carry out the elimination of tumors needs to be revisited. Further understanding the biological attributes of pre-mNK cells which confers them this prominent anti-tumoral potential may improve the design of cancer therapies. A case in point, the presence of a putative human equivalent of pre-mNK cells is positively associated with improved disease outcome in patients affected by refractory solid tumors (32). We herein review the origin of the controversy with regards to the lineage origin and function of pre-mNK cells. In addition, we present the anti-tumoral activity of pre-mNK cells in line with their new mNK-cell precursor function, as well as discuss the identification and biological attributes of the suggested human cellular equivalent.

## Pre-mNK Cells as Part of the NK Lineage

**Figure 1:**
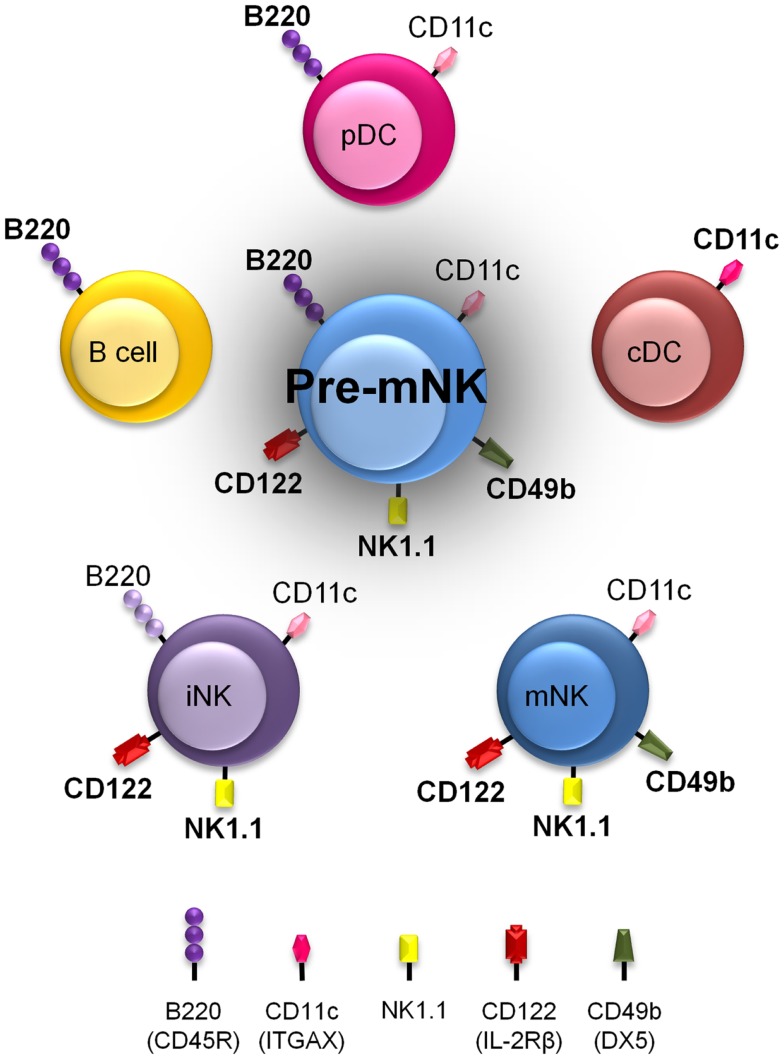
**Pre-mNK cells share phenotypic expression with a variety of other immune cells**. Murine immune cell types harboring cell surface antigens also present on pre-mNK cells are depicted. The intensity in color represents the level of expression. Note that a combination of at least three cell surface antigens, namely CD11c, B220, and CD49b, must be used to clearly distinguish pre-mNK cells from other immune cell types. This shared phenotypic character of pre-mNK cells increases the risk of potential cellular contaminants during the isolation process.

Pre-mNK cells, for their initial name “IKDC,” were first considered as a new DC subset (21, 22). Initial comparative gene expression profile arrays, ultrastructure analysis with electron microscopy, and evaluation of many cell surface markers by flow cytometry suggested a close phenotypic relationship between pre-mNK cells and plasmacytoid DC (pDC) (21, 33) (Figure 1). However, it was subsequently shown that pre-mNK cells represent a unique cell subset more closely related to NK cells (26–28) (Table 1). For one, both mNK and pre-mNK cells are dependent on the Id-2 transcription factor, whereas, in stark contrast, overexpression of Id-2 inhibits pDC differentiation (34, 35). Also, NK cells and pre-mNK cells are absent in Il15^−/−^, Il15ra^−/−^, Rag2^−/−^Il2rg^−/−^, and Rag2^−/−^Il15^−/−^ mice, highlighting their common dependency on IL-15 for differentiation (26, 28, 36). Moreover, it was found that the CD11c^low^ B220^+^ cell surface phenotype was not exclusive to pDC and pre-mNK cells. Indeed, upon *in vitro* activation, NK cells can also acquire the expression of both CD11c and B220 antigens, as well as the expression of several additional cell surface antigens previously thought to specifically distinguish pre-mNK cells from NK cells, namely CD69, CD86, MHCII, FasL, and CD44 (28, 37–40). Furthermore, *in vitro* activated NK cells, as for pre-mNK cells, produce high levels of IFN-γ and exhibit an enhanced cytolytic potential relative to unstimulated NK cells (26, 28, 41). Finally, a parallel can be drawn between pre-mNK cells and the CD56^bright^ NK-cell subset in humans, which has been reported to produce vast amounts of IFN-γ and has also been shown to express MHC II, at least in some experimental settings (7–10, 42, 43). Therefore, these observations strongly suggest that pre-mNK cells are not closely related to pDC. Rather, they appear to represent a subset of NK cells likely to have been recently activated.

**Table 1:** **Properties of pre-mNK cells relative to pDC and NK cells**.

	pre-mNK cells	pDC	mNK cells	Activated NK cells	
**DIFFERENTIATION**					
Id-2	Yes	No	Yes	Yes	
IL-15	Yes	No	Yes	Yes	
γc Cytokines	Yes	No	Yes	Yes	
Flt3L	+	+++	++	
PU.1	No	Yes	No	No	
RelB	Yes	Yes	No	No	
IRF8	Partially	Yes	No	No	
**FUNCTION**					
IFN-γ production	+++	−	+	+++	
IL-12 production	+	++	−	−	
IFN-α production	−	+++	−	−	
Killing mechanism	TRAIL and NKG2D-dependent	No	Perforin/granzyme-dependent	Perforin/granzyme-dependent	
Cytotoxic activity	+++	−	+	+++	
Antigen presentation	+	+	−	+	
**ANTIGENIC PHENOTYPE**				*In vivo*	*In vitro*
CD11c	Low	Low	No/low	No/low	Low
B220	High	High	−	−	High
CD49b	+	−	+	+	+
CD122	+	−	+	+	+
NK1.1	+	−	+	+	+
CD43	+	−/+	+++	+++	+++
Ly108	+++	−/+	−	−	−
CD27	High	−	Low/high	Low/high	Low/high
CD11b	Low/high	−	Low/high	Low/high	Low/high
MHC II	+ (in LNs)/−(in spleen)	+	−	+	+
CD69	+	−	−	+	+
CD86/CD80	+ (in LNs)/−(in spleen)	+	−	−	−
**CHARACTERISTICS**					
Proliferation	++++	+	+	++	

## Pre-mNK Cells as Part of the NK-Cell Differentiation Pathway

Pre-mNK cells exhibit similar phenotypic and functional attributes to *in vitro* activated mNK cells. Hence, our group recently designed experiments to address the *in vivo* biological relationship between pre-mNK cells and mNK cells (30). We first showed that pre-mNK cells are not merely activated mNK cells. Indeed, upon *in vivo* activation with either anti-CD40 or poly I:C, mNK cells did not yield cells carrying the pre-mNK cell phenotype. Instead, we observed that, upon *in vivo* transfer, pre-mNK cells rapidly lose B220 expression and exhibit a parallel increase in the expression of cell surface antigens associated with NK-cell maturation, ultimately acquiring the phenotype of mNK cells. In contrast to the *in vitro* results which suggest that pre-mNK cells are activated mNK, the *in vivo* data demonstrate that pre-mNK cells are precursors to mNK cells.

The apparent discrepancy between the phenotype and function of pre-mNK cells described in both the *in vitro* and *in vivo* setting can likely be explained by variations in the experimental conditions. Firstly, NK cells sorted for *in vitro* culture comprise a pool of both pre-mNK cells and mNK cells which are subject to non-physiological stimuli such as high doses of IL-2. These conditions may favor the survival of pre-mNK cells *in vitro*, allowing for an accumulation of B220^+^ CD49b (DX5)^+^ NK cells over time. This is rather unlikely as pre-mNK cells were shown to rapidly undergo apoptosis upon *in vitro* culture (27). Alternatively, B220 expression may be artificially up-regulated on mNK cells upon exposure to non-physiological stimuli in the *in vitro* setting. It remains to be seen whether B220^+^ mNK cells generated upon *in vitro* culture are equivalent to pre-mNK cells obtained *in vivo*. Secondly, upon *in vivo* transfer, sorted B220^−^ mNK cells did not acquire a pre-mNK cell phenotype in response to either anti-CD40 or poly I:C treatment. Admittedly, it is possible that other *in vivo* stimuli may allow mNK cells to acquire the pre-mNK cell phenotype. For instance, imatinib mesylate (IM) and IL-2 or IL-15 trans-presentation, which increase the proportion of pre-mNK cells *in vivo* (22, 36, 44), may facilitate mNK cells to acquire the pre-mNK cell phenotype. Altogether, the *in vitro* and *in vivo* data suggest that the pre-mNK cell phenotype may be acquired through different means. Regardless, *in vivo*, pre-mNK cells are precursors to mNK cells as they exhibit a close transcriptome relationship to the first stages of mNK cell differentiation and they can effectively generate mNK cells upon *in vivo* transfer (30).

**Figure 2:**
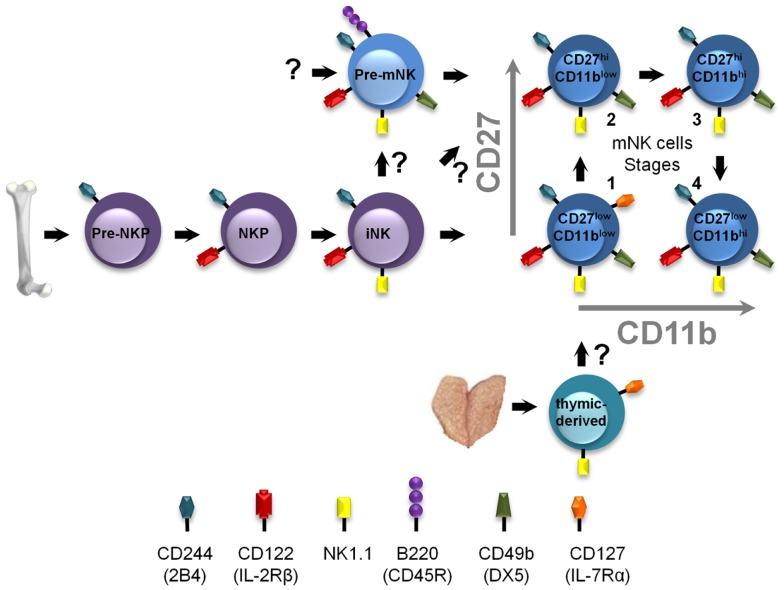
**Positioning pre-mNK cells in the NK-cell differentiation pathway**. NK-cell differentiation is a multi-step process, wherein cellular intermediates can be distinguished based on the acquisition and loss of the expression of specific cell surface antigens, in mice. Pre-NKP cells represent the earliest defined mNK cell-committed progenitor. Here, pre-mNK cells are positioned as a cellular intermediate following iNK cells and preceding mNK cells. Question marks indicate differentiation steps that remain to be experimentally verified. The thymic-derived pathway is also depicted.

Mature NK cells are derived from hematopoietic stem cells in the bone marrow which undergo a series of specific and highly guided differentiation events to eventually yield functional mNK cells (Figure 2). Interestingly, prior studies identified an NK-cell precursor population in the bone-marrow expressing a phenotype similar to pre-mNK cells, namely B220^+^ CD19^−^ CD43^+^ CD24^−^ BP-1 (Ly-51)^−^ cells (45). Although the level of CD11c expression had not been assessed, these cells likely define what is now known as pre-mNK cells, suggesting that pre-mNK cells are also found in the bone marrow. The earliest identified NK cell-committed progenitor are termed pre-NKP cells and arise from the common lymphoid progenitors (CLP) (46). Importantly, these cells have yet to express CD122 (IL-2Rβ), suggesting that this early NK-cell differentiation event is independent of the IL-15 cytokine (46). Pre-NKP cells subsequently generate NKP which differentiate into iNK cells. iNK cells express NK1.1 and gradually acquire CD49b expression as they differentiate into mNK cells. Interestingly, a small proportion of iNK cells express B220 suggesting that this marker is slowly acquired en route to becoming an mNK cell (28). Based on their phenotypic characterization, the CD11c^low^ B220^+^ CD49b^+^ pre-mNK cells are likely to immediately follow the iNK cell stage and, as such, define a late intermediary stage in mNK cell differentiation (Figure 2). The functional maturation of mNK cells can be further subdivided into four stages according to the relative expression of CD27 and CD11b (47), namely stage 1 CD11b^low^CD27^low^, stage 2 CD11b^low^CD27^high^, stage 3 CD11b^high^CD27^high^, and stage 4 CD11b^high^CD27^low^. Still, whether pre-mNK cells present an obligatory or an alternative stage in mNK cells differentiation needs to be resolved. Of interest, while iNK cells produce stage 1 mNK cells, pre-mNK cells appear to differentiate directly into stage 2 mNK cells (30, 47). Therefore, pre-mNK cells may reflect an alternate differentiation pathway arising from iNK cells which can either generate stage 1 mNK cells or pre-mNK cells. Alternatively, pre-mNK cells have been proposed to arise directly from L-selectin progenitors (LSP), while mNK cells were mostly produced from CLP and ELP (34). Pre-mNK cells may altogether define a novel alternate pathway for mNK cell differentiation (Figure 2).

The most striking feature distinguishing pre-mNK cells from mNK cells is the expression of B220. Interestingly, there is evidence that B220 (an alternatively spliced and heavily glycosylated product of *Cd45*) contributes toward defining the number of mNK cells in secondary lymphoid organs, as well as their ability to produce cytokines. Indeed, NK-cell numbers are increased in CD45-deficient mice, and CD45-deficient NK cells show a selective impairment in ITAM-based receptor cytokine production (48–50). Hence, it is tempting to suggest that lack of B220 expression deregulates pre-mNK cells, thereby promoting an altered NK-cell differentiation pathway. More studies are required to assess how pre-mNK cells and how B220 contribute toward defining the size of the NK-cell niche.

Interestingly, thymic NK-cell differentiation is defined as a distinct pathway generating mNK cells in secondary lymphoid organs (51, 52). These thymic-derived mNK cells can be distinguished based on the expression of CD127 (IL-7Rα) and GATA-3 (51) (Figure 2). Notably, neither pre-NKP nor pre-mNK cells seem to be implicated in this pathway; intrathymic injections of pre-NKP cells do not generate mNK cells, whereas pre-mNK cells are virtually undetectable in the thymus and do not express CD127 (21, 46). Still, most stage 1 mNK cells in the secondary lymphoid organs express CD127 and are absent in Rag-deficient mice (30, 47, 51). Together, these results suggest that most stage 1 mNK cells may be derived from thymic precursors. The relationship between the cellular intermediates of each of these NK-cell differentiation pathways has yet to be fully elucidated. Moreover, the type of mNK cells generated from each of these distinct pathways, as well as the specific cellular intermediates, such as CD127^+^-thymic-derived NK cells and pre-mNK cells, may play specific roles in the immune response.

## The Role of Pre-mNK Cells in the Anti-Tumoral Immune Response

Apart from defining an intermediate cell type in the NK-cell differentiation pathway, pre-mNK cells have been primarily studied for their remarkable anti-tumoral potential, which was first uncovered by the group of Zitvogel (22). They demonstrated that pre-mNK cells, and not conventional mNK cells, are responsible for the anti-tumoral response after a treatment with IM in combination with IL-2 (22). Indeed, the adoptive transfer of IM and IL-2-stimulated pre-mNK cells dramatically impaired melanoma tumor outgrowth in immunodeficient (Rag2^−/−^Il2rg^−/−^) mice (22). Interestingly, a similar treatment composed of IM and IL-2 increased the proportion and number of a putative human equivalent to pre-mNK cells, namely HLA-DR^+^ NK cells, in cancer patients and was associated with a better prognostic (32, 53). Additional studies using distinct tumor models and different mouse strains corroborated the remarkable anti-tumoral potential of murine pre-mNK. Notably, the injection of bone-marrow (BM)-derived pre-mNK cells inhibited syngeneic tumor growth in C57BL/6 and beige mice (23).

The anti-tumoral potential of pre-mNK cells is mediated in part by their propensity to effectively secrete elevated quantities of cytopathic cytokines relative to mNK cells (22, 24). Indeed, IFN-γ production by pre-mNK cells can be triggered by IL-12, IL-15, IL-18, and/or various combinations of these cytokines (21, 26, 54). Other factors such as CPG ODN, poly I:C, TLR agonists can also promote IFN-γ production by pre-mNK cells (24, 54). Moreover, tumor cells expressing NKG2D ligands can induce IFN-γ production by pre-mNK cells (23). Other than IFN-γ, pre-mNK cells have been reported to also produce more TNF-α than mNK cells (26). Of note, pre-mNK cells were previously shown to produce IFN-α (21), but this was not confirmed (26, 54). It was proposed that the initial preparation of pre-mNK cells may have been contaminated with pDC which bear a similar phenotype to pre-mNK cells (Figure 1) and are known to produce vast amounts of IFN-α in response to various stimuli (26, 54). Overall, pre-mNK cells have been mostly studied for their ability to produce more IFN-γ than mNK cells (22).

**Figure 3:**
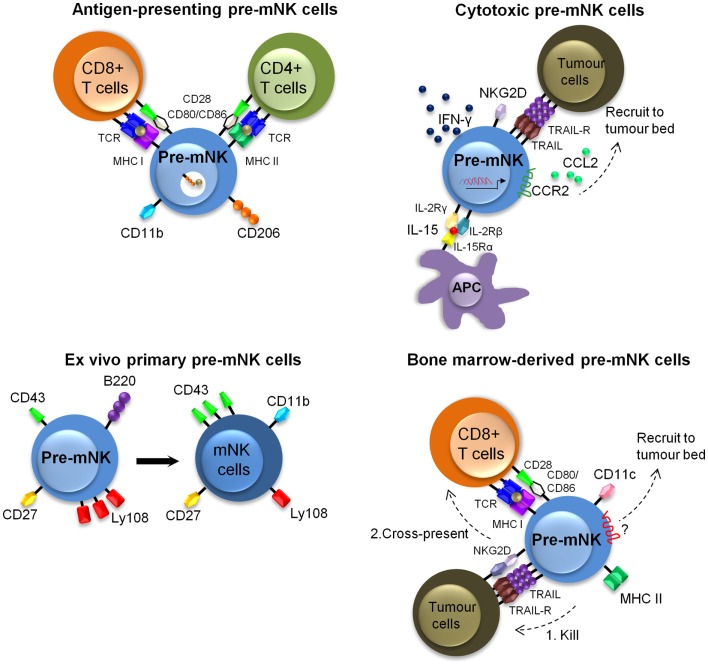
**Described murine phenotypes of pre-mNK cells**. This figure illustrates the different described phenotypes of pre-mNK cells. The top left image represents pre-mNK cells which have the capacity to present antigens to both CD4^+^ and CD8^+^ T cells. Antigen-presenting pre-mNK cells express CD11b and CD206, a mannose receptor implicated in the uptake of antigens. The top right image depicts cytotoxic pre-mNK cells obtained through the trans-presentation of IL-15. IL-15 promotes pre-mNK cells to actively transcribe several genes, thus enhancing IFN-γ production, cytolytic potential, and upregulating the expression of chemokines receptors on pre-mNK cells which facilitates the recruitment at tumor sites. The bottom left image portrays *ex vivo* isolated primary pre-mNK cells, which express high levels of Ly108 and low levels of CD43. Primary pre-mNK cells acquire an mNK cell phenotype upon *in vivo* transfer. The bottom right image illustrates that bone-marrow pre-mNK cells can be recruited to the tumor environment by an unidentified mechanism. Upon killing of a tumor cell, these bone-marrow pre-mNK cells are licensed to cross-present tumor antigens to T cells, initiating the adaptive immune response.

Additional *in vitro* and *in vivo* manipulations of pre-mNK cells have revealed that the cytotoxic activity toward tumoral cells and the antigen-presentation potential to T cells are likely to be uncoupled. It appears that activation of pre-mNK cells with cytokines primes them for enhanced cytolytic potential, whereas interaction with at least some tumoral cell types promotes their antigen-presentation potential (Figure 3). For instance, IL-15 significantly contributes to the anti-tumoral potential of pre-mNK cells, as demonstrated by the decrease of pre-mNK cell effectiveness in Il15^−/−^ mice (55). Enhancing the *in vivo* expression of IL-15 by hydrodynamic injection of a IL-15-expressing cassette also potentiates the number of pre-mNK cells as well as enhances their cytolytic activity (44) (Figure 3). Activated pre-mNK cells have the capacity to kill a murine lymphoma cell line (YAC-1), an MCMV protein expressing cell line (Ba/F3-m157) as well as a melanoma murine tumor cell line (B16F10) (21, 22). The cytotoxic activity of pre-mNK cell is dependent on NKG2D expression and is mediated by TRAIL receptors, in contrast to mNK cells, which preferentially use the perforin/granzyme pathway (22, 36). In addition, *in vitro* IL-15-stimulated pre-mNK cells express unique biological functions and these are not shared by B220^−^ mNK cells (36). Indeed, in contrast to mNK cells, IL-15-stimulated pre-mNK cells show an improved response to type 1 IFN and IL-2 facilitating their migration into tumor beds in a CCL2-dependent manner (36, 55). Moreover, IL-15-stimulated pre-mNK cells acquire resistance to TGF-β induced immunosuppression (36). However, IL-15-stimulated pre-mNK cells lose the capacity to induce MHC class I or II-restricted T cell activation *in vitro*. Altogether, these results indicate that high doses of IL-15 is an efficient means to expand anti-tumoral pre-mNK cells, while inhibiting their antigen-presentation potential.

In contrast, the antigen-presentation potential of *ex vivo* isolated pre-mNK cells appears to be restricted to CD11b^+^ pre-mNK cells (56) (Figure 3). Specifically, the CD11b^+^ pre-mNK cells have the capacity to directly prime naive CD4^+^ T cells or cross-present soluble antigens to naïve CD8^+^ T cells in a CD206 (mannose receptor C type 1, MRC1) and B7-dependent manner. Importantly, the antigen-presentation potential of pre-mNK cells was revealed in this study upon exposure of pre-mNK cells to tumoral cells.

Similarly, the antigen-presentation potential of pre-mNK cells was shown to be potentiated in pre-mNK cells arising from a BM-derived *in vitro* cell culture exposed to tumor cells (Figure 3). Indeed, to investigate the anti-tumoral role of BM-derived-DC, Anderson’s group studied pre-mNK cells present in the BM-DC preparation, likely arising in the culture due to the physiological production and trans-presentation of IL-15 by BM-DC in response to GM-CSF (57, 58). The pre-mNK cells accounted for the anti-tumor activity against 76-9:C23 alveolar rhabdomyosarcoma, as removing pre-mNK cells lead to the loss of the tumoricidal activity (23). Importantly, this approach generated vast numbers of viable BM-derived pre-mNK cells, allowing their specific investigation in both *in vitro* and *in vivo* settings. To that effect, the adoptive transfer of BM-derived pre-mNK cells could inhibit 76.9 tumor growth in B6 mice but not in immunodeficient tumor-bearing host mice showing that BM-derived pre-mNK cells are dependent on the presence of the adaptive immune system for tumor rejection, again supporting their role in antigen-presentation (23, 59). These results contrast with those showing anti-tumoral activity of pre-mNK cells in Rag2^−/−^Il2rg^−/−^ mice carrying established melanomas, suggesting that BM-derived pre-mNK cells may have different migratory or functional properties to that of pre-mNK cells isolated from a spleen (22). Alternatively, alveolar rhabdomyosarcoma or melanoma may induce distinct biological responses from pre-mNK cells. In a subsequent study, BM-derived pre-mNK cells were shown to migrate out of tumor beds toward tumor draining lymph nodes, where they expressed MHC II and costimulatory molecules (59). Altogether, Anderson’s group propose a model where BM-derived pre-mNK cells migrate to the tumor site to lyse tumor cells, take up released tumor antigens, and then present these antigens in the draining lymph nodes. This model is in agreement with the first publication on pre-mNK cells by Housseau’s group showing that pre-mNK cells have specialized function in specific locations (21).

## *In vivo* Investigations of Pre-mNK Cells in Non-Tumoral Immunological Responses

The immunological potential of pre-mNK cells has also been investigated in the context of viral responses and autoimmunity, extending the therapeutic potential of pre-mNK cells to other clinical settings.

To our knowledge, only two studies have attempted to unravel the role of pre-mNK cells in the context of viral responses. First, the human immunodeficiency virus (HIV) was shown to induce cells bearing phenotypic and functional similarity to mouse pre-mNK cells (60). Yet, their role in controlling the viral response has not been investigated. Second, in a mouse model of influenza, pre-mNK cells were shown to exhibit similar functional properties to mNK cells in regulating the immune response in the lung. Both pre-mNK cells and mNK cells presented viral antigens to CD8^+^ but not CD4^+^ T cells and simultaneous depletion of both pre-mNK and mNK cells using anti-NK1.1 antibody inhibited the expansion of viral-specific CD8^+^ T cells but had no effect on viral clearance (29). Interestingly, in support of a role for pre-mNK cells in presenting antigens to T cells in the context of infection, cytomegalovirus-infected fibroblasts were also shown to increase MHC II expression on pre-mNK cells as well as cross-present antigens to CD8 T cells (61). This finding is reminiscent of a study with human NK cells, where exposure to infected cells increases their antigen-presentation potential to T cells (9). Still, additional studies of pre-mNK cells in infectious models based on mNK cell-dependent viral clearance are needed to unravel the specific role of pre-mNK cells in viral responses. As such, it would be of interest to verify if, in the context of chronic viral infections, pre-mNK cells can replenish an exhausted NK-cell pool.

Pre-mNK cells were also shown to modulate autoimmune responses. The first evidence for a role of pre-mNK cells in preventing autoimmune responses was obtained in a virally induced autoimmune-diabetes model (62), adding credence to a potential anti-viral role for pre-mNK cells. Specifically, a bitypic NK/DC population, similar in phenotype to pre-mNK cells, was shown to prevent autoimmune diabetes in the RIP-LCMV mouse model, where diabetes is induced upon LCMV viral infection (62). Notably, the tolerogenic anti-CD40L (CD154) treatment increased the proportion and function of the bitypic NK/DC, which produce high levels of IFN-γ, exhibit an impressive cytotoxic activity, and demonstrate proficient antigen-processing and presentation to T cells. In this setting, it remains to be seen whether the bitypic NK/DCs prevent autoimmune diabetes by disrupting the ongoing autoimmune or viral response. The second indirect evidence in support of a role for pre-mNK cells in preventing autoimmune diabetes arose from an immunogenetic study, wherein it was shown that pre-mNK cell number is low in autoimmune-diabetes prone NOD mice relative to other non-autoimmune-prone inbred mouse strains (63). The low number of pre-mNK cells in NOD mice is linked to a small genetic region on distal end of mouse chromosome 7. This genetic interval had previously been associated to diabetes resistance and inflammatory responses (63–65). Interestingly, NOD.Lc7 mice, congenic for the distal end of mouse chromosome 7 are resistant to autoimmune diabetes and exhibit a restored number of pre-mNK cells in secondary lymphoid organs. Hence, it is tempting to suggest that pre-mNK cells confer autoimmune resistance in the NOD.Lc7 strain. A third study providing additional evidence that pre-mNK cells play a protective role in autoimmune diseases exploited the murine experimental autoimmune encephalomyelitis (EAE) model of multiple sclerosis (66). In this study, pre-mNK cells pre-treated with a tolerizing agent had the capacity to significantly lower the clinical scores of EAE as well as to contribute to EAE remission (66). The induction of tolerance was mediated by the ability of pre-mNK cells to kill activated CD4^+^ T cells and mature DCs as well as to recruit T regulatory cells to the CNS (66). This study was the first to describe a potential biological pathway by which pre-mNK cells may contribute to immune tolerance. Although few studies have investigated the potential role of pre-mNK cells beyond their anti-tumoral response, we believe that the clearer definition of pre-mNK cells as precursors of mNK cells will prompt additional investigation of pre-mNK cells in various immunological settings.

## Key Outstanding Questions

“Interferon-producing killer dendritic cells” have been redefined as NK-cell precursors and, consequently, they have been renamed pre-mNK cells (30, 31). A potentially equivalent cell type has also been identified in humans and, in both mice and humans, pre-mNK cells display a potent anti-tumoral potential (22, 32, 56, 59). In light of these recent findings, here are the key outstanding questions with regards to pre-mNK cell biology and their therapeutic potential.

1. Are pre-mNK cells part of an alternate mNK cell differentiation pathway? Pre-mNK cells are able to generate mNK cells. However, the step preceding pre-mNK cells has not been defined. Additional experiments are required to determine whether iNK cells can generate pre-mNK cells or whether pre-mNK cells arise from a different pathway.
2. What is the nature of the mNK cells produced from pre-mNK cells? The mNK cells generated from pre-mNK cells may exhibit a distinct anti-tumoral potential in comparison to total mNK cells, which also comprise mNK cells derived from iNK cells as well as from the thymus. In agreement with functional segregation of mNK cells based on their origin, it should be noted that thymic-derived mNK cells specialize in cytokine production (51, 52). The functional attributes of mNK cells derived from pre-mNK cells will need to be assessed. One could speculate that mNK cells generated from pre-mNK cells could bear enhanced cytotoxic properties and be particularly geared toward anti-tumoral responses (51). In support of this view, the anti-tumoral potential of *in vivo* transferred pre-mNK cells was mostly TRAIL-dependent (22). A direct comparison of thymic-derived, iNK-derived and pre-mNK-derived mNK cells will help determine whether the alternate differentiation pathways lead to a common mNK cell fate or to distinct mNK cell phenotypes, each exhibiting unique functional characteristics.
3. Is the heightened anti-tumoral potential of pre-mNK cells relative to mNK cells due to their capacity to generate vast numbers of mNK cells? Pre-mNK cells as precursors to mNK cells have the capacity to generate mNK cells. Yet their efficiency at generating mNK cells *in vivo* in the context of an inflammatory response has not been documented. Pre-mNK cells may be more effective than mNK cells on a per cell basis simply due to the propensity of pre-mNK cells at generating a larger number of effective mNK cells *in vivo*. Interestingly, the suggested human equivalent to pre-mNK cells, HLA-DR^+^ NK cells, is highly proliferative suggesting that they are poised to efficiently yield high numbers of mNK cells (43). The *in vivo* experiments comparing the potential of pre-mNK cells and mNK cells at inducing tumor regression were monitored over several days, providing sufficient time for pre-mNK cells to potentially generate large quantity of mNK cells. However, the ability of pre-mNK cells to generate mNK cells was not quantified over time in the tumoral settings. Therefore, although pre-mNK cells proliferate rapidly and are able to generate mNK cells, their proficiency to do so in an inflammatory setting has yet to be assessed (30).
4. Is the heightened anti-tumoral potential of pre-mNK cells relative to mNK cells due to their unique functional attributes? Pre-mNK cells themselves may exhibit unique immunological properties conferring them a greater anti-tumoral potential on a per cell basis relative to mNK cells. Indeed, in contrast to the non-inflammatory setting where pre-mNK cells rapidly acquire an mNK cell phenotype, pre-mNK cells maintain their phenotype in an inflammatory context (30). Inflammation may abrogate pre-mNK cell differentiation into mNK cells, contributing to an accumulation of cells exhibiting a pre-mNK cell phenotype. As pre-mNK cells exhibit a phenotype reminiscent of *in vitro* activated mNK cells and demonstrate high levels of IFN-γ production as well as most prominent cytotoxic activity *in vitro* in short term assays, it is tempting to suggest that pre-mNK cells play a key role in the regulation of the anti-tumoral response (22, 27, 28). Clearly, additional studies are required to fully grasp how pre-mNK cells mediate their remarkable anti-tumor functions. A combination of their ability to generate a large number of mNK, as well as their unique functional attributes, likely contributes to their enhanced anti-tumoral activity.
5. What are the conditions which are permissive for pre-mNK cells to present antigens to T cells? Pre-mNK cells were originally shown to process and present antigens to T cells (21). This observation, as for IFN-α production, was not reproducibly observed by others, again suggesting that the original cellular preparations were contaminated with pDC (26, 54). However, using an intricate *in vitro* system, pre-mNK cells were shown to exhibit efficient antigen-presenting potential when they were co-incubated in the presence of tumoral cells (59). This antigenic presentation potential of pre-mNK cells is dependent on their cytolytic potential, namely TRAIL and NKG2D (21–23, 59). In line with these results, the contact of pre-mNK cells with tumor cells increases MHC II expression via an autocrine production of IFN-γ and also enhances the expression of costimulatory molecules (56). Together, these studies, which thoroughly compared the antigen-presentation potential of pre-mNK cells, NK cells, and conventional DCs to T cells, support the unique antigen-processing and presentation potential for pre-mNK cells (56, 59, 61). Still, it could be argued that, as pre-mNK cells exhibit a phenotype more closely related to pDC rather than conventional DC (Figure 1), it may be relevant to revisit some of these findings in relation to pDC. Regardless of the debate, the antigen-presentation potential of pre-mNK cells has been most consistently observed when pre-mNK cells are exposed to tumoral cells. How and which tumoral environment enhances the antigen-presentation potential of pre-mNK cells remain to be established.
6. Do mouse pre-mNK cells and their recently described putative human equivalent, HLA-DR+ NK cells, truly represent equivalent cell types? No unique markers (whether it be a cell surface marker, a functional property or a transcription factor necessary for their differentiation) can currently be used to define pre-mNK cells. Thus, to establish their similitude, comparative analyses of mouse and human pre-mNK cells must be performed. Importantly, the potential of human pre-mNK cells at generating mNK cells should be addressed.
7. Can the putative human pre-mNK cells be specifically used as a cellular therapeutic? There are currently no known factors that uniquely promote pre-mNK cell numbers *in vivo*. Although IL-15 is efficient, it also enhances mNK cells as well as memory T cells. Pre-mNK cells are difficult to isolate, due to their low number, and show poor viability *in vitro*. As to be expected with the discovery of a novel cell types and notwithstanding their prominent anti-tumoral potential, there are still many challenges ahead before pre-mNK cells can effectively be used in therapy. The most promising current therapeutic avenue shown to possibly promote pre-mNK cells, which correlates with improve disease outcome, remains the combination of IM and IL-2 (32, 53).

## Concluding Remarks

Over the past years, conflicting results on the phenotype and function of pre-mNK cells highlighted the challenges associated with defining a cell type present in low number in lymphoid organs. As the attention focused in defining the lineage origin of pre-mNK cells, it shifted away from better understanding the striking anti-tumoral potential of pre-mNK cells. Indeed, murine pre-mNK cells and the recently proposed human homolog exhibit a prominent anti-tumoral activity. As the field of pre-mNK cell biology moves forth we anticipate the development of novel therapeutic approaches in the treatment of cancer and eventually also in the treatment of other pathologies.

## Conflict of Interest Statement

The authors declare that the research was conducted in the absence of any commercial or financial relationships that could be construed as a potential conflict of interest.
